# SLAMF7 (CD319) enhances cytotoxic T-cell differentiation and sensitizes CD8^+^ T cells to immune checkpoint blockade

**DOI:** 10.3389/fimmu.2025.1654374

**Published:** 2025-08-20

**Authors:** Jan-Erik Sander, Irina Han, Lisette Fickenscher, Jörg-Peter Schmidt, Hartmut Kroll, Tereza Vosikova, Martin Durisin, Holger Lingel, Monika C. Brunner-Weinzierl

**Affiliations:** ^1^ Department of Experimental Pediatrics, University Hospital, Otto-von-Guericke-University, Magdeburg, Germany; ^2^ Institute for Transfusion Medicine Dessau, Red Cross Blood Transfusion Service NSTOB, Dessau, Germany; ^3^ Department of Otorhinolaryngology, Head and Neck Surgery, University Hospital, Otto-von-Guericke-University, Magdeburg, Germany

**Keywords:** SLAMF family, T-cell differentiation, cytotoxicity, costimulation, cancer, immunotherapy, tumor rejection, head and neck squamous cell carcinoma (HNSCC)

## Abstract

Tumors frequently evade immune destruction by impairing cytotoxic CD8^+^ T-cell responses, highlighting the need for strategies that restore T-cell functionality. Here, we identify SLAMF7 (CD319) as a key enhancer of human CD8^+^ T-cell responses against tumors. SLAMF7 expression is induced by pro-inflammatory signals such as IL-12 and CD28 co-stimulation. Agonistic SLAMF7 signaling, in synergy with TCR activation, is able to strongly induce T-cell activation and clonal expansion, a finding consistently observed in CD8^+^ T cells from healthy adults as well as derived from blood and tumor-draining lymph nodes of patients with head and neck squamous cell carcinoma (HNSCC). Moreover it drives a distinct differentiation programme characterized by elevated expression of key transcription factors Eomes and T-bet, leading to increased production of effector molecules such as Interferon γ, Granzyme B and Perforin. In contrast to CD28 costimulation, SLAMF7 activation also promotes serial killing potential via BTLA induction. In antigen-specific human models, SLAMF7 activation boosts CD8^+^ T-cell responses against the tumor-associated antigen NY-ESO-1, a key target in several cancers including HNSCC. Moreover, combining SLAMF7 activation with PD-1/PD-L1 immune checkpoint blockade synergistically enhances cytokine release and cytotoxic potential, highlighting its potential to overcome immunosuppression and reinvigorate antitumor immunity.

## Introduction

Restoring effective T-cell responses is central to tumor immunosurveillance and cancer control. Tumors employ immune escape mechanisms - including secretion of TGF-β and expression of PD-L1 - to inhibit CD8^+^ T-cell effector function (1). Immune checkpoint blockade (ICB) with antibodies targeting CTLA-4 or PD-1/PD-L1 can reverse this suppression and reinvigorate anti-tumor immunity (2). ICB has become a key component of treatment across multiple cancer types. In head and neck squamous cell carcinoma (HNSCC), PD-1 inhibitors such as Pembrolizumab and Nivolumab are approved as first-line therapies for recurrent or metastatic disease and combinational therapy is arising (3–5). However, therapeutic efficacy of ICB in boosting tumor rejection is limited by immune-related adverse events (e.g., colitis, hepatitis, pneumonitis, endocrinopathies) and low rates of durable responses (6–8). Accordingly, there is a pressing need to develop next-generation immunotherapies that extend clinical benefit and limit treatment-associated toxicity.

Analysis of downstream targets of the inhibitory molecule CTLA-4 on CD8^+^ T cells revealed potential targets to improve or provide an alternative to established immune checkpoint therapies (9). One of the potential targets is the self-ligating receptor SLAMF7 (CD319), a member of the Signaling Lymphocyte Activation Molecule (SLAM) family expressed on hematopoietic cells (10–15). Through its cytoplasmic immunoreceptor tyrosine-based switch motifs (ITSMs), it has been proposed to recruit several Src homology-2 (SH-2) domain-containing adapter proteins such as EAT-2, SHP-1 and SHP-2, SHIP1, Csk, Fyn or PLC-γ (14–16). On NK cells, SLAMF7 is known to promote activation and degranulation, but also mediate inhibitory signals in the absence of the adaptor EAT-2 (13, 14, 16). Due to its high expression on myeloma cells monoclonal antibodies have been developed to target SLAMF7 as a tumor antigen, leading to NK-cell mediated antibody-dependent cellular cytotoxicity (ADCC) (17–19). Further it has been shown that SLAMF7 promotes phagocytosis of cancer cells by macrophages through interaction with Mac-1 (20).

In T cells, SLAMF7 is predominantly expressed on cytotoxic CD8^+^ T cells and on a subset of cytolytic CD4^+^ T cells (21, 22). Its engagement has been shown to restore effector function in dysfunctional CD8^+^ T cells in systemic lupus erythematosus, and to enhance cytotoxicity in tumor-specific CD4^+^ T cells upon agonistic stimulation (23, 24). However, in cancer-related contexts, SLAMF7 expression on CD8^+^ T cells is also associated with T cell exhaustion and a suppressive phenotype (25, 26). In a murine model, SLAMF7 engagement during CD8^+^ T-cell priming was shown to integrate environmental cues into acquisition of cytotoxic effector functions (15). These findings underline the ambivalent role of SLAMF7 in T-cell biology, ranging from immune activation to dysfunction, depending on the cellular and environmental context.

In this study, we aimed to resolve this ambiguity by identifying upstream signals that regulate SLAMF7 expression in human CD8^+^ T cells and by assessing whether SLAMF7 co-stimulation enhances or impairs CD8^+^ T-cell responses. Our findings provide insights into the contextual role of SLAMF7 in CD8^+^ T cells and explore its potential as a target to modulate T-cell function for cancer immunotherapy.

## Materials and methods

### Human samples

Leukocyte reduction cones from healthy adult donors were obtained from the University Blood Bank Magdeburg (12/2023–03/2024) and the German Red Cross Dessau (04/2024–11/2024). Peripheral blood and lymph node tissue from HNSCC patients were provided by the Department of Otorhinolaryngology, University Hospital Magdeburg. Written informed consent was obtained from all participants in accordance with the Declaration of Helsinki, and the study was approved by the local ethics committee of the University of Magdeburg (OVGU) (Certificate 53/19).

### Enrichment of cells

PBMCs were isolated by density gradient centrifugation using Pancoll (PAN Biotech). CD8^+^ T cells and CD14^+^ monocytes were purified by magnetic cell separation (Miltenyi Biotec) according to the manufacturer’s protocol, reaching routinely purities of ≥98%. Tumor-draining lymph nodes were minced and digested overnight at 37°C in RPMI containing 1 mg/ml collagenase, 5% heat-inactivated FCS, and Penicillin/Strepto-mycin. Single-cell suspensions were filtered and processed as above for CD8^+^ T-cell isolation. CD8^+^ T cells were cultured in X-VIVO 15 medium (Lonza) supplemented with 10 ng/ml IL-12 (Proteintech), unless stated otherwise. For stimulation, 5 µm sulfate polystyrene microspheres (Thermo Fisher) were coated with antibodies or fusion proteins at 1 × 10^8^ microspheres/ml in DPBS.

### T-cell stimulation

Microspheres were loaded with 1 µg/ml αCD3 ab (clone HIT3α, BioLegend) in combination with either 3 µg/ml αSLAMF7 ab (clone 162.1, BioLegend), 3 µg/ml SLAMF7-Fc fusion protein (R&D Systems), or 3 µg/ml IgG2b isotype control ab (BioLegend). αCD3 ab (1 µg/ml) plus αCD28 ab (0.5 µg/ml, BioLegend) served as positive control (**Supplementary Table 1A**). Cells were stimulated at a 5:4 cell-to-microsphere ratio. For antigen-specific activation microspheres were coated with 2 µg/ml recombinant HLA-A2:Ig fusion protein (DimerX, BD Biosciences) together with either 3 µg/ml αSLAMF7 or IgG2b isotype control ab. To load HLA molecules, these microspheres were pulsed with 1 µg/ml CEFX or NY-ESO-1 peptides (JPT Peptide Technologies), washed and used in T-cell stimulation assays. IL-2 (10 ng/ml) was added on day 3. Cells were stimulated at a 3:2 cell-to-microsphere ratio.

### Checkpoint blockade following SLAMF7 activation

For immune checkpoint blockade following SLAMF7 activation, CD14^+^ monocytes and CD8^+^ T cells were isolated from PBMCs as described above. Monocytes were cultured with 10 ng/ml CSF-1 and pulsed on day 3 with 1 µg/ml NY-ESO-1. CD8^+^ T cells were pre-activated for 4 days using microspheres (see above), then incubated for 15 min with 10 µg/ml αPD-1 and 10 µg/ml αPD-L1 or isotype control (BD Biosciences) (**Supplementary Table 1A**). Subsequently, 1 × 10^5^ CD8^+^ T cells were co-cultured with antigen-loaded monocytes at a 2:3 ratio for 24 h. On day 5, T cells were transferred to ELISpot plates (CTL Europe) for analysis.

### ImmunoSpot analysis

Secretion of IFNγ and Granzyme B was analyzed using a double-color ELISpot assay (ImmunoSpot^®^ IFNγ/Granzyme B, CTL Europe). After five days of microsphere-based stimulation (polyclonal or antigen-specific) or APC restimulation, cells were washed (DPBS, Thermo Fisher Scientific) and cultured in serum-free CTL-Test™ Medium (CTL Europe) supplemented with 2 mM L-glutamine. Cells were plated on PVDF multiscreen plates (Merck Millipore) pre-coated with αIFNγ and αGranzyme B capture antibodies (24 h, 4 °C). The assay was performed according to the manufacturer’s protocol. Secreted IFNγ and Granzyme B were detected using αIFNγ-FITC and biotinylated αGranzyme B, followed by αFITC-HRP and Streptavidin-AP, and developed using corresponding chromogenic substrates (all CTL Europe). IFNγ spots appear red, Granzyme B spots blue. Quantification was performed with an ImmunoSpot S6 analyzer (CTL, USA). Besides individual ELISpot images included in the main figures, two representative ELISpot assays are provided in the supplement (**Supplementary Figure S1**, **Supplementary Table S2**).

### Cytokine-multiplex assay

For quantification of the secreted cytokines IL-6, IL-10, Perforin and Fas ligand (FasL), supernatants were taken from the cell cultures after five days of polyclonal microsphere stimulation and analyzed by a cytokine multiplex assay (LEGENDPlex, BioLegend). The Assay was performed according to the manufacturer’s instructions. Cytometric measurements were performed on a 4-Laser LSRFortessa X-20 (BD Biosciences) and results were analyzed with LEGENDplex Data Analysis Software Suite (Qognit) (**Supplementary Figure S2**).

### Flow cytometric analysis

Flow cytometry was used to assess surface markers, intracellular proteins (**Supplementary Table 1B**), and T-cell proliferation. CD8^+^ T cells were labeled with CellTrace™ Violet (Thermo Fisher Scientific) to track proliferation. Surface staining was performed for 15 min at 4 °C in the dark using fluorochrome-conjugated antibodies against BTLA, CD8, PD-L1 (1 µg/ml), CD25, CD69, CD137, PD-1, and SLAMF7 (clone 235614) (0.5 µg/ml); CD107a staining (1 µg/ml) was performed for 4 h. For antigen-specific assays, HLA-A02:Ig dimers loaded with peptides of interest were used; donors were pre-typed by SBT or screened using an anti-HLA-A02 antibody (1 µg/ml, BioLegend). For intracellular staining of Eomes, T-bet, and Granzyme B, cells were fixed on day 5 in 4% formaldehyde (20 min, 37 °C), briefly frozen at –20 °C, and permeabilized in 90% methanol (30 min, –20 °C). Fixed cells were stained with fluorescent antibodies (1 µg/ml) for 1 h at 4 °C in the dark. Data acquisition was performed on a 4-laser LSRFortessa X-20 (BD Biosciences) and analyzed using FlowJo software (BD Biosciences). In addition to the flow cytometric images included in the main figures, gating strategy and representative flow plots for BTLA, PD-1 and Eomes are provided in the supplement (**Supplementary Figure S3**).

### Statistical analysis

Statistical analysis was performed using Prism 10 (Dotmatics). Normality was assessed by Shapiro-Wilk test and Q-Q plots. Based on distribution, either parametric (t-test, one-way ANOVA) or non-parametric (Wilcoxon) tests were applied. Outliers (maximum one per group) were excluded using Grubbs’ test (α = 0.01). Data points represent independent biological replicates; bars show mean ± SD. Statistical significance is indicated as *p < 0.05, **p < 0.01, ***p < 0.001, ****p < 0.0001. For multiple comparisons, p-values were adjusted using the Bonferroni-Holm test (see **Supplementary Table 3**).

## Results

### Proinflammatory signals induce SLAMF7 on human CD8^+^ T cells

**Figure 1:**
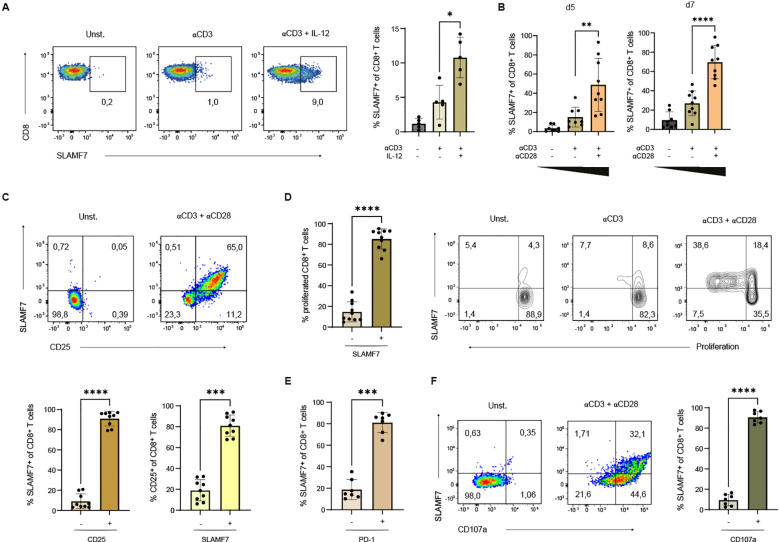
Expression of SLAMF7 on human CD8^+^ T cells. **(A)** CD8^+^ T cells were activated with αCD3 coupled microspheres with or without IL-12 (10 ng/ml) as indicated. The frequency of SLAMF7^+^ CD8^+^ T cells was analyzed after 5 days of stimulation (unstimulated control is shown). One sample has been normalized to the mean of αCD3 engagement. **(B)** Frequency of SLAMF7^+^ CD8^+^ T cells was analyzed after 5 and 7 days of stimulation with αCD3 coupled microspheres depending on the presence of costimulatory αCD28 signal. **(C–F)** Expression of SLAMF7 was correlated with different surface markers as well as proliferation after 5 days of stimulation with αCD3/αCD28 coupled microspheres. **(C)** Frequency of CD25^+^ CD8^+^ T cells within the SLAMF7^+^ T-cell population in comparison to CD25^-^ SLAMF7^+^ CD8^+^ T cells (brown) and frequency of SLAMF7^+^ within CD25^+^ CD8^+^ T cells was compared to SLAMF7^+^ CD25^+^ CD8^+^ T cells (yellow). **(D)** The proliferation of CTV-labeled CD8^+^ T cells stimulated with αCD3-coupled microspheres with or without αCD28 was analyzed by flow cytometry. Frequency of proliferating CD8^+^ T cells expressing SLAMF7 in comparison to SLAMF7^-^ CD8^+^ T cells. **(E)** Comparison of PD-1 expressing CD8^+^ T cells to PD-1 negative/low CD8^+^ T cells within the SLAMF7^+^ CD8^+^ T-cell population. **(F)** SLAMF7^+^ CD8^+^ T cells were analyzed for the surface expression of CD107a. Data points represent independent biological replicates with mean and SD. *p < 0.05; **p < 0.01; ***p < 0.001; ****p < 0.0001; p-values were calculated using one-way ANOVA with Tukey´s multiple comparisons test **(A, B)** or two-tailed paired t-test **(C–F)**.

To characterize the signals that drive SLAMF7 expression on the surface of human CD8^+^ T cells, we exposed CD8^+^ T cells to different stimuli. Since full-fledged T-cell activation depends on TCR activation, co-stimulatory signals and inflammatory cytokines (27), we aimed to investigate whether these factors influence the expression of SLAMF7. First, the effect of the Tc1-inducing cytokine IL-12 was analyzed (28) (**Figure 1A**). CD8^+^ T cells were stimulated with anti-CD3 coupled microspheres and either IL-12 was added to the cell culture medium or not. Resting T cells did hardly express SLAMF7. The Tc1-inducing cytokine IL-12 more than doubled the frequency of CD8^+^ T cells expressing SLAMF7 from 4% after CD3 engagement to approximately 11% after CD3 engagement in the presence of IL-12 (**Figure 1A**, right). Next, the effect of co-stimulation on SLAMF7 expression of CD8^+^ T cells was investigated (**Figure 1B**). For this purpose, CD8^+^ T cells were stimulated with anti-CD3 plus co-stimulatory anti-CD28 engagement or anti-CD3 plus isotype control in the presence of IL-12. At both time points, co-stimulatory CD28 signaling resulted in up to three times higher frequency of SLAMF7-expressing CD8^+^ T cells compared to anti-CD3 engagement alone. Comparing day 5 with day 7 after beginning of the stimulation, the frequency of SLAMF7^+^ CD8^+^ T cells increased with both anti-CD3 stimulation alone and with the co-stimulatory signal. The maximum frequency of SLAMF7^+^ CD8^+^ T cells was reached after 7 days of stimulation with anti-CD3 plus anti-CD28 coupled microspheres, with an average of 70% SLAMF7-expressing CD8^+^ T cells.

To elucidate the characteristics of CD8^+^ T cells expressing SLAMF7, inhibitory and activation associated surface molecules were analyzed for co-expression with SLAMF7. Therefore, CD8^+^ T cells were stimulated with anti-CD3/anti-CD28 microspheres for 5 days. SLAMF7 was found to be highly co-expressed with the α-chain of the IL-2 receptor (CD25) (**Figure 1C**, upper panel), which can be considered as a surrogate marker for T-cell proliferative potential as IL-2-signals trigger the proteolytic degradation of constitutively expressed cell cycle inhibitors (29, 30). Within the SLAMF7^+^ T-cell population, around 90% of T cells expressed CD25. Vice versa, analyzing all CD25^+^ T cells revealed that 80% of CD25^+^ CD8^+^ T cells express SLAMF7 (**Figure 1C**, lower panel), stressing the point that SLAMF7 is strongly associated with activated T-cells having a proliferative potential. To assess SLAMF7 expression during T-cell proliferation, CD8^+^ T cells were labeled with the fluorescent dye CTV prior stimulation (**Figure 1D**). It revealed that almost all proliferating CD8^+^ T cells were SLAMF7^+^. Furthermore, SLAMF7 was found to be already expressed on a significant number of CD8^+^ T cells within the initial generation, demonstrating that no full mitotic cell cycle is required for SLAMF7 expression (**Figure 1D**). When comparing co-expression of SLAMF7 with PD-1 on CD8^+^ T cells after 5 days of anti-CD3/anti-CD28 engagement, cytometric analysis revealed that 80% of SLAMF7^+^ T cells expressed PD-1 (**Figure 1E**), making it reasonable that SLAMF7^+^ T cells are susceptible to inhibitory signals (31). To investigate whether there is a link between SLAMF7 and the cytotoxic potential of CD8^+^ T cells, the expression of surface CD107a, a molecule exposed at the surface during T-cell degranulation, was analyzed (32, 33). The results demonstrated that approximately 90% of SLAMF7^+^ CD8^+^ T cells were positive for CD107a (**Figure 1F**).

### Concomitant engagement of SLAMF7 and TCR/CD3 leads to the expansion of human CD8^+^ T cells which have potential cytotoxic effector functions

**Figure 2:**
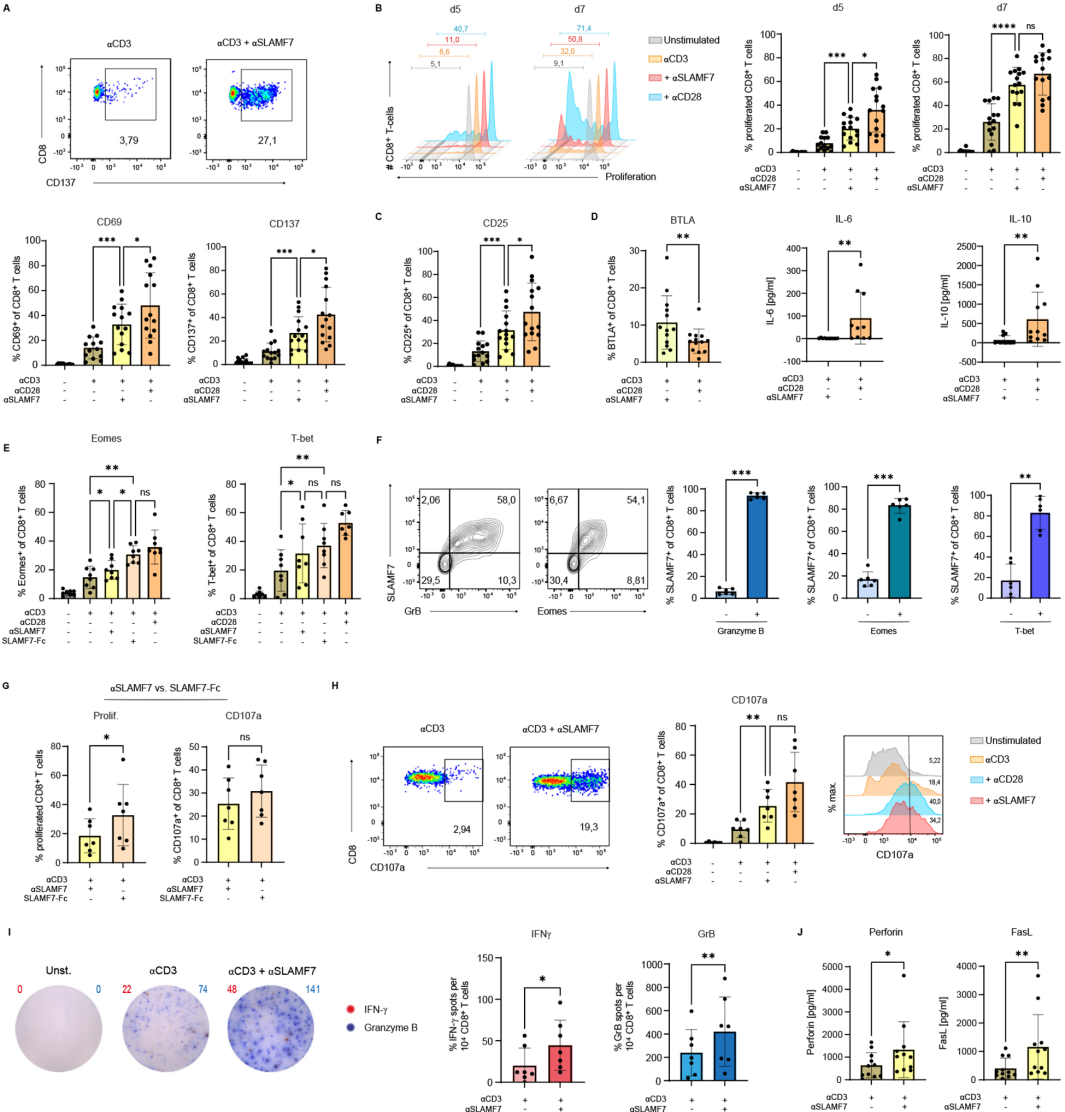
SLAMF7 engagement leads to activation, expansion and differentiation of cytotoxic CD8^+^ T cells. **(A)** CD8^+^ T-cell surface expression of the activation associated molecules CD69 and CD137 was analyzed on day 5 after engagement of SLAMF7 via agonistic αSLAMF7 coupled to αCD3 microspheres compared to αCD3 alone, αCD3/αCD28 activation or unstimulated condition. **(B)** Proliferation of CTV-labeled CD8^+^ T cells was analyzed on day 5 and day 7 of stimulation with αCD3/isotype, αCD3/αSLAMF7 or αCD3/αCD28 coupled microspheres **(C)** as well as the surface expression of the IL-2R (CD25) on day 5 post stimulation. **(D)** Frequency of BTLA^+^ CD8^+^ T cells analyzed by flow cytometry and the cytokine concentrations of IL-6 and IL-10 in the cell-culture supernatants were compared between αCD3/αSLAMF7 and αCD3/αCD28 coupled microsphere stimulation on day 5. **(E)** Frequency of Eomes^+^ respectively T-bet^+^ CD8^+^ T cells was analyzed after 5 days of stimulation with αCD3/αSLAMF7 or αCD3/SLAMF7-Fc fusion protein coupled microspheres. As references αCD3/αCD28 or αCD3/isotype coupled microspheres were used, likewise unstimulated T cells. **(F)** Expression of SLAMF7 was correlated with the T-cell function promoting transcription factors Eomes and T-bet as well as intracellular presence of Granzyme B on day 5 after stimulation with αCD3/αCD28 coupled microspheres. **(G)** Comparison of αCD3/αSLAMF7 and αCD3/SLAMF7-Fc fusion protein engagement in terms of T-cell proliferation (CTV-dye) and frequency of CD107a expressing CD8^+^ T cells 5 days post stimulation. **(H)** Frequencies of CD107a^+^ CD8^+^ T cells were compared after 5 days of stimulation with αCD3/αSLAMF7, αCD3/isotype or αCD3/αCD28 coupled microspheres. **(I)** IFNγ/Granzyme B double color ELISpot assay was conducted 5 days after stimulation with αCD3/αSLAMF7, αCD3/isotype or αCD3/αCD28 coupled microspheres, the number of spot-forming units was than compared. **(J)** Levels of Perforin and FasL were analyzed in the cell culture supernatants by a cytokine-multiplex assay after 5 days of either αCD3/Isotype or αCD3/αSLAMF7 engagement. Data points represent independent biological replicates with mean and SD. *p < 0.05; **p < 0.01; ***p < 0.001; ****p < 0.0001; p-values were calculated using one-way ANOVA (mixed effects analysis) with Tukey`s multiple comparisons test **(A, B, C, E, H)**, paired t-test (**(D)** (BTLA), **(E, G, I)** (GrB)) and Wilcoxon-test (**(D)** (IL-6, IL-10), **(I)** (IFNγ), **(J)**).

After characterizing SLAMF7 expression on human CD8^+^ T cells, we next aimed to specifically assess the functional consequences of SLAMF7 activation in the context of concurrent TCR stimulation. To this end, SLAMF7-specific antibodies (ab) were co-immobilized with anti-CD3 on microspheres to provide defined, simultaneous engagement of SLAMF7 and the TCR. Anti-CD3 coupled together with isotype control served as a negative control. For comparative purposes, SLAMF7 co-stimulation was evaluated alongside classical co-stimulation via CD28, using anti-CD3/anti-CD28-coated microspheres (**Figure 2A**, upper panel). Given the central role of IL-12 in CD8^+^ T-cell responses and its ability to induce SLAMF7 surface expression (**Figure 1A**), the cytokine was subsequently added to the cell culture. First, expression of the activation-associated molecules, CD69 and CD137 were analyzed (**Figure 2A**) (34–37). Frequencies of CD8^+^ T cells expressing these molecules were significantly upregulated by two to three times after 5 days of stimulation with anti-CD3/anti-SLAMF7 compared to CD3 engagement alone. While SLAMF7-mediated co-stimulation led to approximately 30% of CD8^+^ T cells expressing CD69 or CD137, co-stimulation by CD28 led to a frequency of 45% CD69^+^ or CD137^+^ of CD8^+^ T cells. Furthermore, SLAMF7 engagement in concordance with anti-CD3 stimulation was able to induce proliferation of CD8^+^ T cells (**Figure 2B**). Flow cytometric analysis demonstrated an increase from about 10% proliferating T cells with anti-CD3 engagement alone to about 25% divided T cells after anti-CD3 engagement together with anti-SLAMF7 on day 5 after beginning of the stimulation. This effect was also observed on day 7, where SLAMF7 engagement in concordance with anti-CD3 more than doubled the frequency of expanded T cells compared to anti-CD3 activation alone, to an average of approximately 60% proliferated T cells. However, T cells co-stimulated with anti-CD28 proliferated faster than cells receiving an agonistic SLAMF7-specific ab, but unlike on day 5, no significant difference between the two conditions was observed on day 7 (**Figure 2B**). As IL-2 signals are prerequisite for differentiation and CD8^+^ T-cell proliferation, surface expression of CD25 was analyzed (**Figure 2C**) (30, 38). CD25 was shown to be upregulated three-fold by anti-CD3/anti-SLAMF7 compared to anti-CD3/isotype activation, while engagement of CD3/CD28 molecules resulted in a four-fold increase in the frequency of CD25^+^ CD8^+^ T cells. The findings, when considered as a whole, indicate that SLAMF7, when in conjunction with TCR, has the capacity to induce the differentiation of effector cells, as well as the expression of CD25 and the subsequent proliferation of human CD8^+^ T cells.

As we have demonstrated that CD28-costimulation increases the frequency of SLAMF7-expressing CD8^+^ T cells (**Figure 1B**), we investigated whether engaging SLAMF7 together with CD3 can induce the surface expression of SLAMF7 on human CD8^+^ T cells (**Supplementary Figure S4A**). Here we show that stimulation with anti-CD3/anti-SLAMF7 coupled microspheres significantly increased the frequency of SLAMF7-expressing CD8^+^ T cells compared to stimulation with anti-CD3/isotype coupled microspheres. However, stimulation of T cells by anti-CD3/anti-CD28 coupled microspheres was superior regarding the induction of SLAMF7-surface expression compared to CD3/SLAMF7 engagement.

Since SLAMF7 engagement seems to serve as a co-stimulatory receptor similar to CD28 in terms of T-cell proliferation and activation, the next aim was to investigate whether the two types of co-activation, anti-SLAMF7 and anti-CD28, lead to a different type of T-cell differentiation program. Engagement of SLAMF7 in conjunction with engaging CD3 on human CD8^+^ T cells, using specific antibodies coupled to microspheres, led to a significantly higher frequency of T cells expressing the B- and T-lymphocyte attenuator (BTLA) compared to activation with anti-CD3/anti-CD28 (**Figure 2D**). Indicating that although SLAMF7 seems to exert co-stimulatory functions like CD28, the differentiation of the T cells appears to be divergent. This aberrant T-cell differentiation was also observed by analyzing the cytokines IL-6 and IL-10 in the supernatants of the T-cell cultures using a cytokine multiplex assay (LegendPlex, BioLegend) (**Figure 2D**, **Supplementary Figure S2**).

In order to gain a more detailed insight into the intracellular processes that take place in T cells during TCR/SLAMF7 activation, the CD8^+^ T-cell function promoting transcription factors Eomes and T-bet were analyzed, as well as the intracellular presence of Granzyme B, the latter indicating their potential cytotoxic capacity (**Figure 2E**) (39–44). For this purpose, T-cell stimulation was performed not only with an agonistic SLAMF7-specific ab, but also using a recombinant SLAMF7-Fc fusion protein immobilized on microspheres. As the fusion protein engages SLAMF7 via its natural binding interface, it offers a potentially more physiological mode of activation that could reveal distinct aspects of SLAMF7 signaling. For both Eomes and T-bet, engagement of SLAMF7 molecules via an agonistic ab resulted in a significantly higher frequency of T cells containing these molecules compared to CD3 engagement alone (**Figure 2E**). This effect, at least for Eomes, was even stronger by applying the SLAMF7-Fc fusion protein instead of the SLAMF7-specific ab. When comparing the frequency of Granzyme B containing T cells following either anti-CD3/isotype or anti-CD3/anti-SLAMF7 activation, no significant difference was observed (**Supplementary Figure S4B**). However, stimulation with the recombinant SLAMF7-Fc fusion protein resulted in a significantly higher rate of Granzyme B positive T cells than CD3 activation alone (**Supplementary Figure S4B**). To investigate whether there is a link between T cells expressing the transcription factors Eomes or T-bet and the presence of SLAMF7 on the T-cell surface, correlation analysis was performed after 5 days of anti-CD3/anti-CD28 microsphere stimulation (**Figure 2F**). More than 80% of the SLAMF7-positive T cells were found to co-express Eomes, as was also the case for T-bet (**Figure 2F**, right). The surface expression of SLAMF7 was also analyzed in relation to intracellular Granzyme B. Here the correlation was even stronger, almost all SLAMF7-positive T cells contain Granzyme B (**Figure 2F**, middle), highlighting the cytotoxic potential of T-cells expressing SLAMF7 (**Figure 2F**).

Having identified the superiority of the fusion protein over the agonistic SLAMF7-specific ab regarding the induction of Eomes and Granzyme B, further investigation on the SLAMF7-Fc fusion protein was conducted (**Figure 2G**). Frequency of proliferated CD8^+^ T cells increased from around 20% with the agonistic SLAMF7-specific ab up to 35% by stimulation with the SLAMF7-Fc fusion protein, after 5 days (**Figure 2G**, left). To assess the impact of SLAMF7 activation on the differentiation of cytotoxic CD8^+^ T cells, surface-expressed CD107a, an indicator of released cytotoxic vesicles, was analyzed (32, 33). No significant difference was observed in the frequency of CD8^+^ T cells expressing CD107a between stimulation with anti-SLAMF7 ab and recombinant SLAMF7 (**Figure 2G**, right). In contrast, stimulation with anti-CD3 and anti-SLAMF7 ab significantly increased the frequency of CD107a^+^ T-cells compared to CD3 stimulation alone - just as potent as CD3/CD28 engagement (**Figure 2H**). To further strengthen the finding of improved degranulation and cytotoxicity upon CD3/SLAMF7 engagement, the secretion of well-established T-cell effector function markers, IFNγ and Granzyme B was assessed by an ELISpot assay (**Figure 2I**) (45). SLAMF7 co-stimulation significantly increased the number of spot-forming units for both cytokines: Granzyme B spots approximately doubled, and IFNγ spots tripled compared to CD3/isotype stimulation. The cytotoxicity promoting effect of SLAMF7 co-stimulation was further supported by multiplex analysis (LegendPlex), revealing significantly elevated levels of Perforin and soluble FasL in the cell culture supernatants following SLAMF7 co-stimulation compared to CD3 engagement alone (**Figure 2J**) (46–49).

These data demonstrate that agonistic SLAMF7 signals during TCR stimulation not only promotes activation and proliferation of human CD8^+^ T cells, but also drives their differentiation. T cells triggered by SLAMF7-specific ab in conjunction with TCR/CD3 demonstrate increased expression of the transcription factors Eomes and T-bet, which promote CD8^+^ T-cell differentiation and effector function. Thus, resulting in a more effective Tc1- response and improved cytotoxic effector function.

### SLAMF7 engagement enhances CD8^+^ T-cell response to viral and tumor antigens

**Figure 3:**
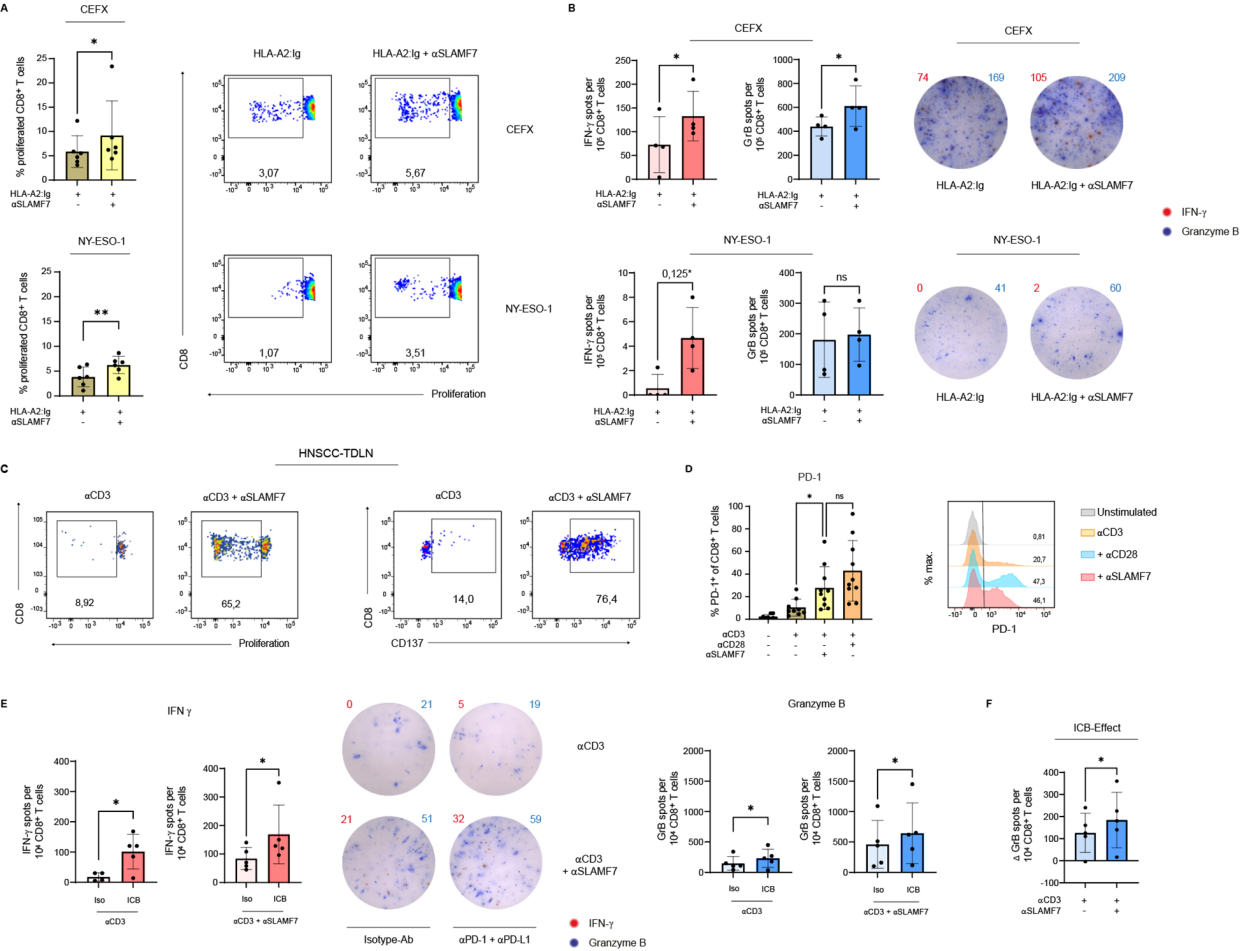
SLAMF7 engagement during antigen-specific CD8^+^ T-cell activation and in combination with αPD-1/αPD-L1 blockade. For antigen specific activation, human CD8^+^ T cells were stimulated with recombinant HLA-A2:Ig fusion protein coupled on microspheres. Either CEFX, a peptide mix of several infectious antigens, or the cancer-testis-antigen NY-ESO-1 was loaded onto the HLA receptor. **(A)** Proliferation of the CTV-labeled antigen-specific activated CD8^+^ T cells was analyzed on day 11 post stimulation, agonistic αSLAMF7 was coupled to the microspheres or not. One sample has been normalized to the mean of TCR engagement only (HLA-A2:Ig/isotype ctrl.). **(B)** Double-color ELISpot assay was performed after 5 days of antigen-specific stimulation to determine the release of IFNγ and Granzyme **(B)** CD8^+^ T cells remained 24h on the ELISpot plate, number of spot forming units was compared between conditions with or without an agonistic SLAMF7 signal. One sample has been normalized to the mean of TCR engagement only, except comparison of Granzyme B - spots after stimulation with NY-ESO-1 loaded microspheres. *corrected p-value after Bonferroni-Holm test (not corrected p=0,0625) **(C)** CD8^+^ T cells isolated from TDLNs from patients with advanced stages of HNSCC were stimulated either with αCD3/isotype or αCD3/αSLAMF7 coupled microspheres. Flow cytometric analysis of the proliferation (CTV-dye) and CD137 surface expression was performed on day 9 post stimulation (one out of two similar experiments shown). **(D)** Frequency of PD-1^+^ CD8^+^ T cells after 5 days of stimulation with αCD3 coupled microspheres with or without αSLAMF7 or αCD28 (unstimulated control is shown). **(E)** To link SLAMF7 activation with an ICB, CD8^+^ T cells were first stimulated with microspheres coated with αCD3 alone or in addition with αSLAMF7 for 4 days, followed by a co-culture with NY-ESO-1 pulsed CD14^+^ APC´s. For immune checkpoint blockade, 10 µg/ml αPD-1 plus 10 µg/ml αPD-L1 were supplemented into the APC/CD8^+^ co-culture, an isotype-ab was used as control. CD8^+^ T cells remained 24h in the co-culture, before secretion of IFNγ and Granzyme B was analyzed by an ELISpot assay. For Granzyme B, one sample has been normalized to the mean of αCD3/isotype activation without secondary ICB. **(F)** Difference of Granzyme B spot forming units between conditions with and without PD-1/PD-L1 blockade was calculated, the resulting ICB-effect was compared between primary αCD3/isotype and primary αCD3/αSLAMF7 engagement. Data points represent independent biological replicates with mean and SD. *p < 0.05; **p < 0.01; p-values were calculated using paired t-test **(A, B, E, F)**, Wilcoxon test (**A, B, E** (Proliferation CEFX, IFNγNY-ESO-1, IFNγICB after SLAMF7)) or one-way ANOVA (mixed effects analysis) with Tukey`s multiple comparisons test **(D)**. ns, not significant.

Having identified the beneficial effects of SLAMF7 activation regarding CD8^+^ T-cell responses in polyclonal activating settings, it was investigated whether these results could be extended to CD8^+^ T-cell responses against viral and also tumor antigens. To this end, a recombinant MHC-I molecule (DimerX I human) was coupled to microspheres and pulsed with either CEFX, a peptide mixture of dominant epitopes of different infectious antigens from common viral pathogens, such as Haemophilus influenza, human herpesviruses, Influenza A etc., or with NY-ESO-1, a tumor antigen from the group of cancer-testis antigens (50). Either an agonistic SLAMF7-specific ab was simultaneously coupled to the antigen-coated microspheres or not. Exposing CD8^+^ T cells from donors being HLA-A*02 carriers, to viral pathogens (CEFX), agonistic SLAMF7 signals almost doubled the frequency of expanded cells from about 5% to 9% (**Figure 3A**, upper panel). Upon presentation of the low-affinity tumor-associated antigen NY-ESO-1 to the CD8^+^ T cells, co-stimulation by SLAMF7 also led to significantly enhanced proliferation. In conjunction with TCR/CD3 engagement, the frequency of proliferating T cells was increased from about 3% without SLAMF7 engagement to about 7% by receiving it (**Figure 3A**, lower panel).

Next, we examined whether SLAMF7 engagement using specific ab is able to enhance the cytotoxic effector function of antigen-specifically stimulated CD8^+^ T cells (**Figure 3B**). Therefore, an ELISpot assay was conducted analyzing secretion per cell of the effector cytokine IFNγ and cytolytic molecule Granzyme B after 5 days of stimulation. It disclosed that engaging SLAMF7 using specific ab on human CD8^+^ T cells during activation with microspheres presenting different infectious antigens (CEFX) via MHC I, results in an increased amount of T cells releasing IFNγ and even Granzyme B. More precisely, SLAMF7 signaling leads to a nearly two-fold increase of cells secreting IFNγ, while for Granzyme B there was an increase of about 40% (**Figure 3B**, upper panel). Regarding NY-ESO-1-specific activation of T cells, there was a tendency (p=0,0625, Bonferroni-Holm corrected p=0,125) for SLAMF7 co-stimulation to promote the release of IFNγ, but not for Granzyme B release (**Figure 3B**, lower panel).

Overall, both the viral peptide mixture CEFX and the cancer-testis antigen NY-ESO-1 showed that additional SLAMF7 signaling using specific ab during antigen-specific activation leads to increased clonal expansion of CD8^+^ T cells. However, investigation of the impact of SLAMF7 on the release of cytotoxic effector cytokines revealed that SLAMF7 has a significant effect in the context of CEFX-specific activation, and a tendency for NY-ESO-1.

### SLAMF7 restore functionality of CD8^+^ T cells from patients with head and neck cancer

Since we figured out that SLAMF7 engagement enhances T-cell responses against the tumor antigen NY-ESO-1 in healthy donors, we wanted to investigate whether this effect is preserved in cancer patients. NY-ESO-1 is re-expressed in various malignancies, e.g. melanoma, head and neck, lung, liver, stomach, and ovarian cancer (51, 52). In our study we focused on patients with advanced stages of HNSCC. CD8^+^ T cells were isolated from tumor-draining lymph nodes (TDLN) and subsequently stimulated with anti-CD3/anti-SLAMF7-coupled microspheres (**Figure 3C**). Here, we demonstrate that SLAMF7 agonism, together with CD3 stimulation, is able to strongly induce proliferation of CD8^+^ T cells from TDLNs, whereas CD3 engagement alone was almost ineffective (**Figure 3C**, left panel). In addition, anti-CD3/anti-SLAMF7 stimulation also increased the frequency of CD8^+^ T cells from TDLNs expressing CD137 compared to anti-CD3/isotype engagement (**Figure 3C**, right panel). This suggests a supportive effect of agonistic SLAMF7 signals on the activation of CD8^+^ T cells from TDLNs of HNSCC patients. Furthermore, anti-CD3/anti-SLAMF7 engagement increased the frequency of CD8^+^ T cells from HNSCC-TDLN expressing CD107a compared to single CD3 activation, indicating a restoration of their cytotoxic function (**Supplementary Figure S5A**). This reinforcing effect of their cytotoxic function was also observed in CD8^+^ T cells isolated from the peripheral blood of a HNSCC patient (**Supplementary Figure S5B**).

### ICB after prior SLAMF7 activation increases cytotoxic potential of human CD8^+^ T cells

Building on the observed enhancement of CD8^+^ T-cell responses by SLAMF7, we next assessed whether co-application of immune checkpoint blockade could synergistically amplify the SLAMF7-mediated effects. As PD-1 is an approved target of ICB we investigated whether SLAMF7 enhances the expression of PD-1 on human CD8^+^ T cells. Indeed, SLAMF7 stimulation together with CD3 activation leads to an increase in PD-1 expressing CD8^+^ T cells from about 10% after CD3 activation alone to about 30% PD-1^+^ CD8^+^ T cells (**Figure 3D**). Of note, the effect was similar as when an effector T cell response was initiated via TCR/CD3 and CD28 engagement. Therefore, an ICB was mimicked by a PD-1/PD-L1 blockade in an APC/CD8^+^ T-cell co-culture to examine whether the effect of SLAMF7 on CD8^+^ T-cell immune responses could be enhanced. To evaluate this in a tumor-specific setting, CD8^+^ T cells pre-activated with anti-CD3/anti-SLAMF7 or anti-CD3/isotype coupled microspheres were co-cultured with CSF-1-matured, NY-ESO-1–pulsed APCs in the presence or absence of PD-1/PD-L1 blockade using specific ab. ELISpot analysis revealed that ICB blockade of PD-1 and PD-L1 significantly enhanced the number of cells releasing IFNγ after prior SLAMF7 activation by a factor of two (**Figure 3E**, right (red)). However, ICB following anti-CD3 engagement of CD8^+^ T cells only was also potent in increasing the absolute numbers of CD8^+^ T cells releasing IFNγ (**Figure 3E**, left (red)). Nonetheless, it should be noted that without ICB the release of IFNγ with anti-CD3 stimulation alone was below the level of T cells receiving an additional SLAMF7 signal using SLAMF7-specific ab. Even after blocking PD-1 and PD-L1 the total amount of IFNγ spot forming units was about 50% higher with primary CD3/TCR and SLAMF7 activation than without SLAMF7 activation (**Figure 3E** (red)). Regarding the release of Granzyme B, PD-1/PD-L1 blockade was also able to increase the number of T cells secreting the cytotoxic effector cytokine after primary TCR/CD3 and SLAMF7 activation, by approximately 50%. For primary CD3 activation alone, immune checkpoint blockade during restimulation with NY-ESO-1 pulsed APCs increased Granzyme B release as well, but it is noteworthy that the level was approximately three times lower than that of T cells receiving CD3 stimulation and SLAMF7 signal (**Figure 3E** (blue)). Remarkably, although the initial level of Granzyme B release after primary SLAMF7 activation was already higher than after CD3 activation alone, the effect of PD-1/PD-L1 blockade was nevertheless stronger in SLAMF7-stimulated CD8^+^ T cells than in T cells that did not receive SLAMF7 signaling prior to ICB (**Figure 3F**).

Taken together, these results demonstrate that SLAMF7 engagement during T-cell activation improves the cytotoxicity of human CD8^+^ T cells against viral and tumor-antigens. Moreover, sequential SLAMF7 engagement followed by ICB improves T-cell responses against the tumor-associated self-antigen NY-ESO-1.

## Discussion

**Figure 4:**
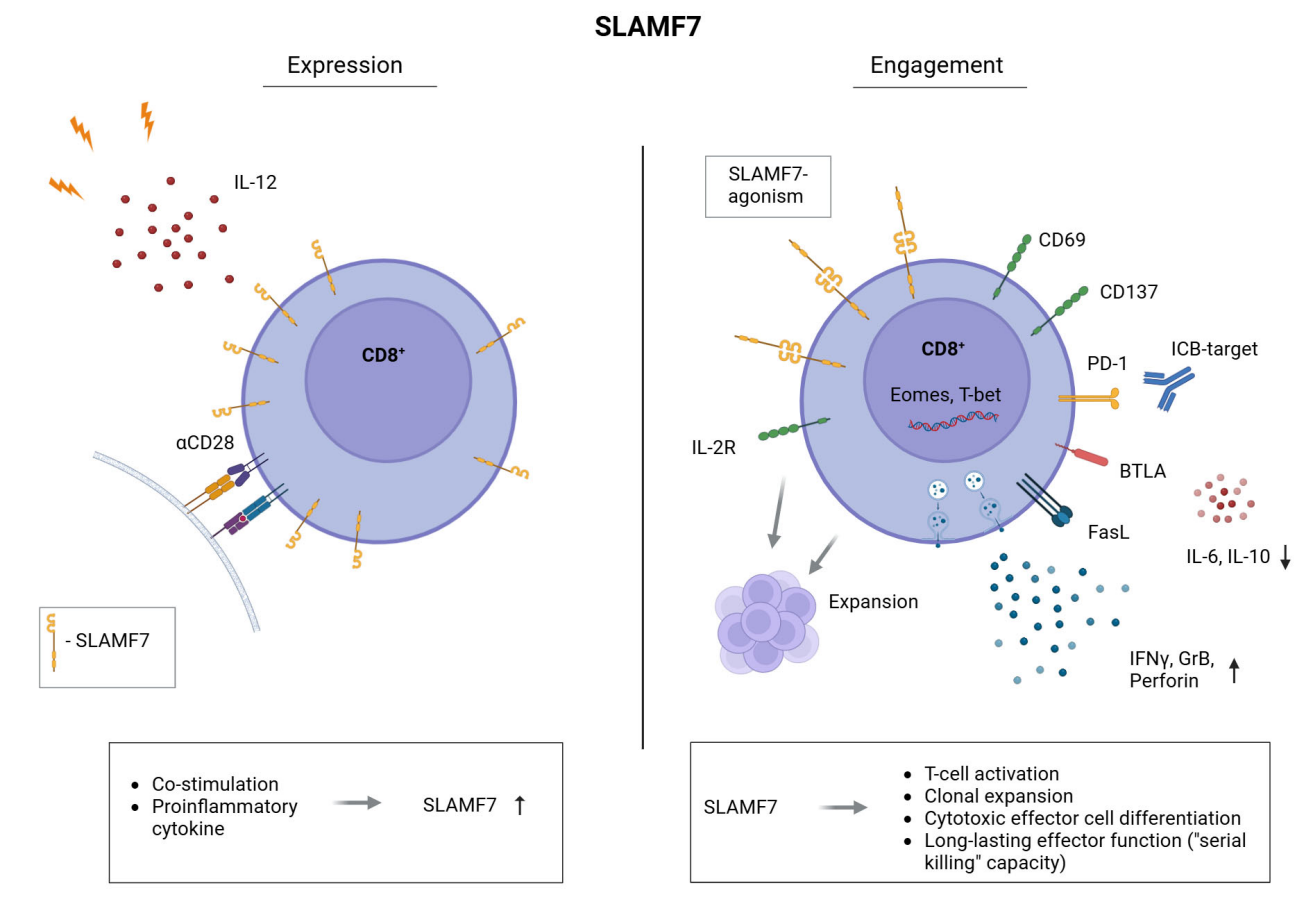
Expression and engagement of SLAMF7 on human CD8^+^ T cells. Frequency of CD8^+^ T cells expressing SLAMF7 on their surface is increased by CD28-co-stimulation and the Th1 inducing cytokine IL-12 (left). CD8^+^ T cells presenting SLAMF7 on their surface are potentially susceptible to receive SLAMF7 signals by SLAMF7-SLAMF7 interaction with other immune cells. Mimicking these interactions by agonistic SLAMF7 antibody or SLAMF7-Fc fusion protein, we uncovered the role of SLAMF7 in human CD8^+^ T cells. Here, we found that SLAMF7 engagement induces T-cell activation (CD69, CD137), the expression of the IL-2R (CD25) as well as their clonal expansion. We demonstrated that SLAMF7 engagement leads to an aberrant T-cell differentiation compared to CD28 co-stimulation, as secretion of immunosuppressive IL-10 as well as IL-6 was downregulated by SLAMF7 compared to CD28-signals. Furthermore, the frequency of BTLA^+^ CD8^+^ T cells was significantly increased following SLAMF7 engagement compared to CD28 co-stimulation. BTLA is associated with a long-lasting effector function and BTLA^+^ TILs show a capacity for “serial killing” of their target cells ^57^. Moreover, T-cell function promoting transcription factors T-bet and Eomes were expressed among a higher frequency of CD8^+^ T cells after engaging SLAMF7. In line, agonistic SLAMF7 signals improve the cytotoxic effector function of CD8^+^ T cells by an enhanced release of effector cytokines (IFNγ, Granzyme B, Perforin) as well as upregulation of the cell death inducing FasL. Finally, combining SLAMF7 engagement with anti-PD-1/anti-PD-L1 checkpoint blockade results in a further boost of cytotoxic T-cell responses.

Our study demonstrates that SLAMF7 co-stimulation enhances activation, proliferation, and cytotoxic differentiation of human CD8^+^ T cells (**Figure 4**), including those from tumor-draining lymph nodes of HNSCC patients. Furthermore, sequential SLAMF7 engagement followed by PD-1/PD-L1 blockade synergistically boosts effector function against the tumor-associated antigen NY-ESO-1. In addition, we uncovered that SLAMF7 is upregulated on CD8^+^ T cells after activation – prior to completion of the first mitotic cell cycle. Therefore, SLAMF7 seems to play an important role in transducing environmental cues into early immune responses (15). Consistent with this, SLAMF7 engagement promoted IFNγ secretion, a cytokine critical for initiating and amplifying immune responses (45). Moreover, SLAMF7 signaling induced CD25 expression, marking the onset of IL-2 responsiveness and clonal expansion. These findings suggest that SLAMF7 not only reflects early T-cell activation, but actively contributes to the transition toward effector differentiation and proliferation.

Here, we demonstrated that agonistic SLAMF7 stimuli provide a potent co-stimulatory signal for CD8^+^ T cells, independent from CD28 - the canonical second signal in the classical two-signal model (53). Beside the promoting effect regarding T-cell activation and clonal expansion, we demonstrated that SLAMF7 activation increased the frequency of T cells expressing BTLA compared to CD28 co-stimulation. This suggests that SLAMF7 engagement supports a less-differentiated CD8^+^ T-cell phenotype with a sustained effector capacity, including “serial killing” (54). Furthermore BTLA expression on CD8^+^ tumor-infiltrating lymphocytes was associated with improved clinical outcome in melanoma (54), aligning with our observation that SLAMF7 co-stimulation supports a less-differentiated, cytotoxic phenotype with sustained effector capacity. In contrast to CD28 signaling, SLAMF7 engagement reduced IL-10 secretion, a cytokine with well-established immunosuppressive functions, as well as IL-6 levels, which has been implicated in inflammation-driven tumor progression (55–58). Therefore, SLAMF7 promotes T-cell responses by providing an “alternative” co-stimulatory signal, with potential benefits beyond CD28-mediated co-stimulation.

SLAMF7 engagement recapitulates several canonical co-stimulatory features of CD28, but these signals appear to be highly dependent on the receptor’s close spatial proximity to the TCR within the immunological synapse (15). As a result, isolated ligation of SLAMF7 — such as through soluble antibodies — is unlikely to initiate productive signaling in CD8^+^ T cells. This contrasts with NK cells, where SLAMF7 signaling is effective even in the absence of synaptic localization (18, 26). From a therapeutic perspective, this spatial dependency introduces a significant challenge: SLAMF7-targeting antibodies, intended to eliminate SLAMF7-expressing tumor cells, may inadvertently bind SLAMF7 on effector CD8^+^ T cells, marking them for Fc-mediated clearance. This off-target effect could diminish anti-tumor immunity, particularly in patients who have not yet accumulated dysfunctional or suppressive SLAMF7^+^ CD8^+^ T cell subsets (25).

Furthermore, SLAMF7-mediated hallmarks that are also elicited by CD28 — such as sustained proliferation — it does so with slower kinetics. Of note, direct comparisons must be interpreted with caution because anti-SLAMF7 antibodies, SLAMF7-Fc fusion proteins, and CD28 ligands differ both in binding affinity and in the conformational changes they impose on their target receptors. However, this delay of SLAMF7 costimulation is not merely quantitative; it coincides with a qualitatively distinct transcriptional programme (e.g. BTLA expression for potential “serial killing”) that skews T cells toward enhanced cytotoxicity, persistence and metabolic flexibility. Consequently, SLAMF7 provides an alternative co-stimulatory pathway that may trade speed for functional breadth, an attribute that could be particularly advantageous in tumor or otherwise immunocompromised microenvironments where CD80/CD86 expression is absent or functionally impaired (1).

Previous studies linked SLAMF7 expression to cytotoxic lineage identity (15, 22). Our data extend this concept by demonstrating that SLAMF7 not only marks, but functionally reinforces cytotoxic programming in CD8^+^ T cells. This was proven by the induction of the T-box transcription factors Eomes and T-bet upon SLAMF7 co-stimulation. Both Eomes and T-bet assume partly complementary functions in the differentiation of naive CD8^+^ T cells into cytotoxic effector cells. Among other functions, they regulate the expression of IFNγ, Perforin and Granzyme B, which are central to the cytotoxic activity of T cells (39, 40, 59–61). In line with this, our data demonstrate that TCR/SLAMF7 engagement leads to increased release of the cytotoxicity-mediating cytokines Perforin, Granzyme B as well as the apoptosis-inducing Fas ligand and the effector molecule IFNγ (41–43, 46–48, 62–65). To be noted, the secretion of these cytokines were assessed in the extracellular matrix, either by a cytokine-multiplex-assay (Perforin, Fas ligand) or by an ELISpot-assay (IFNγ, Granzyme B). Therefore, it can be assumed that the secreted cytokines exert their cytotoxic effects on the affected target cells, e.g. virus-infected or tumor cells, leading to target cell lysis. Further evidence that SLAMF7 co-stimulation leads to enhanced cytotoxic activity of human CD8^+^ T cells was provided by the finding that TCR/SLAMF7 engagement favors the degranulation of CD8^+^ T cells, as assessed by the surface expression of CD107a, which is usually located in the membrane of cytotoxic vesicles and only expressed on the T-cell surface upon degranulation (32, 33). The cytotoxicity promoting effects of SLAMF7-mediated co-stimulation on human CD8^+^ T cells highlights the potential of the SLAMF7-receptor as a therapeutic target to enhance CD8^+^ T cell responses leading to improved elimination of virus-infected or tumor cells.

A limitation of our study is that we stop at the transcription factors T-bet and Eomes without mapping the proximal signals initiated by SLAMF7. Lacking EAT-2 in T cells and with only marginal SAP recruitment, the single ITSM motif is therefore predominantly occupied by SHP-1/2 and other alternative adaptors (14, 16, 66). SHP-2, far from purely representing a phosphatase, can promote ERK- and PI3K-driven proliferation via GRB2/Gab2 scaffolds, thus offering a route to the heightened cell division and transcriptional output we observe (67–70). Complementing this, SILAC interactomics in murine CD8^+^ T cells identified CRK/CRKL and Nck on SLAMF7, pointing to LAT-GRB2 and actin-remodeling modules that could underlie the increased cytotoxicity (15). Downstream, STAT1/3 activity and MAPK pathway could drive T-bet, Eomes, Granzyme B and Perforin, providing a plausible mechanistic bridge between SLAMF7 ligation and the cytolytic programme we report (26, 71). The usage of adaptors is highly context-dependent, shaped by the phosphorylation status of ITSM, adaptor abundance, and competition with other receptors for shared signaling molecules. The next important step is to identify these TCR/SLAMF7-mediated adaptor–kinase combinations that are active in effector, memory and tumor-infiltrating CD8^+^ T-cell subsets.

Apart from that, we found that SLAMF7 agonism induces the expression of PD-1 on CD8^+^ T cells during their effector response. Suggesting that these T cells might be especially susceptible for inhibitory signals provided by interaction with its ligands PD-L1 and PD-L2 (31, 72, 73). However, the frequency of PD-1^+^ T cells remained lower compared to CD3/CD28-activated controls. A previous study reported that T cells activated *in vitro* by SLAMF7 express inhibitory receptors, but differ from terminally exhausted T cells in that they retain the ability to produce cytokines upon re-stimulation (26). Therefore, upregulation of PD-1 via SLAMF7 signaling is more likely part of a natural regulatory feedback mechanism, potentially dampening T-cell activation and function and thereby protecting T-cells from activation-induced cell death (74). This is especially relevant in chronic infections and cancer, where sustained T-cell activation is required, but immune checkpoint molecules such as PD-1 can limit excessive immune responses and prevent damage to healthy tissues (75, 76). In this context, SLAMF7 engagement may induce PD-1 without impairing cytotoxic potential, indicating that co-expression of SLAMF7 and PD-1 may signify a regulated yet functionally competent subset. It would be interesting to further investigate whether these T cells ultimately transition to a classical exhausted state or maintain robust effector capacity.

In our antigen-specific human model, agonistic stimulation of SLAMF7 enhanced clonal expansion in the context of infectious threats as well as for the tumor-associated antigen NY-ESO-1 (51). Together with the improved release of the effector cytokines Granzyme B and IFNγ, at least after exposure to the infectious peptide mixture, this is promising that modifying T cells by SLAMF7 could serve as a novel strategy to fight infectious threats and malignant diseases. As we have shown by CD8^+^ T cells from tumor-draining lymph nodes as well as from the peripheral blood of patients with advanced stages of HNSCC, engaging SLAMF7 is also able to enhance T-cell responses in a presumably immunocompromised environment, where T-cell function is downregulated. This further underlines the potential of SLAMF7 to restore T-cell function in the tumor environment and makes SLAMF7 an interesting agonistic target for immunotherapeutic approaches in solid tumors.

Here, our data reveal that the effects of a primary SLAMF7 activation regarding the secretion of IFNγ and Granzyme B could be further amplified by subsequent ICB when NY-ESO-1 is presented via APCs. Remarkably, the ICB could not only improve the impact of SLAMF7, it also has been observed that the effect of the PD-1/PD-L1 blockade in terms of Granzyme B release was more pronounced in SLAMF7-primed T cells compared to T cells that did not received a SLAMF7 signal prior to ICB. Although we have not formally quantified synergy, the enhanced anti-tumor activity seen with prior SLAMF7 co-stimulation and PD-1/PD-L1 blockade underscores that dual targeting of co-stimulatory and inhibitory pathways is beneficial, irrespective of whether the interaction is strictly additive or synergistic. Therefore, our data suggest that SLAMF7 agonism sensitises CD8^+^ T cells to checkpoint inhibition, supporting a combinatorial strategy that could broaden the efficacy of checkpoint-based tumor immunotherapy.

Overall, this study reports a hitherto unknown role of the self-ligating receptor SLAMF7 on human CD8^+^ T cells. SLAMF7 could be identified to enhance CD8^+^ T-cell responses against infectious but also tumor antigens. Therefore, modifying T cells by their SLAMF7 receptor, e.g. in the sense of an autologous T-cell transfer, might be an effective strategy to enhance tumor immune surveillance (77, 78). Thus, we revealed a putative novel target molecule which has the potential to improve the effectiveness of cancer immunotherapy and, besides combinational therapy, offers an alternative strategy for patients not responding to established checkpoint inhibitors.

## Data Availability

The raw data supporting the conclusions of this article will be made available by the authors, without undue reservation.
