# Supplementary figures and images for: SLAMF7 (CD319) enhances cytotoxic T-cell differentiation and sensitizes CD8+ T cells to immune checkpoint blockade
Source: Front Immunol. 2025 Aug 20;16:1654374. doi: 10.3389/fimmu.2025.1654374 (PMC12405405; doi:10.3389/fimmu.2025.1654374)

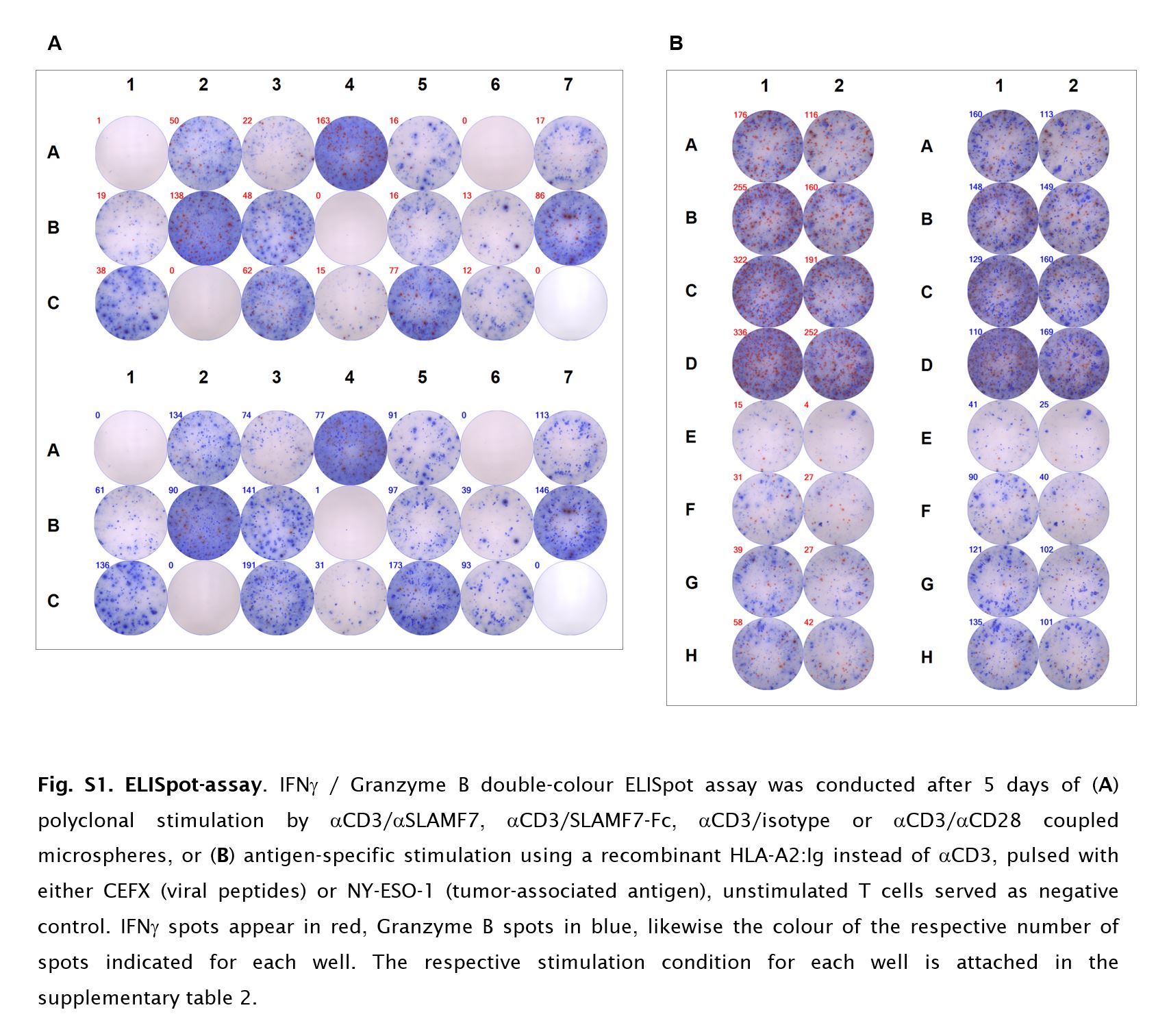

Supplement: Supplementary file 1 [file Image1.jpeg]

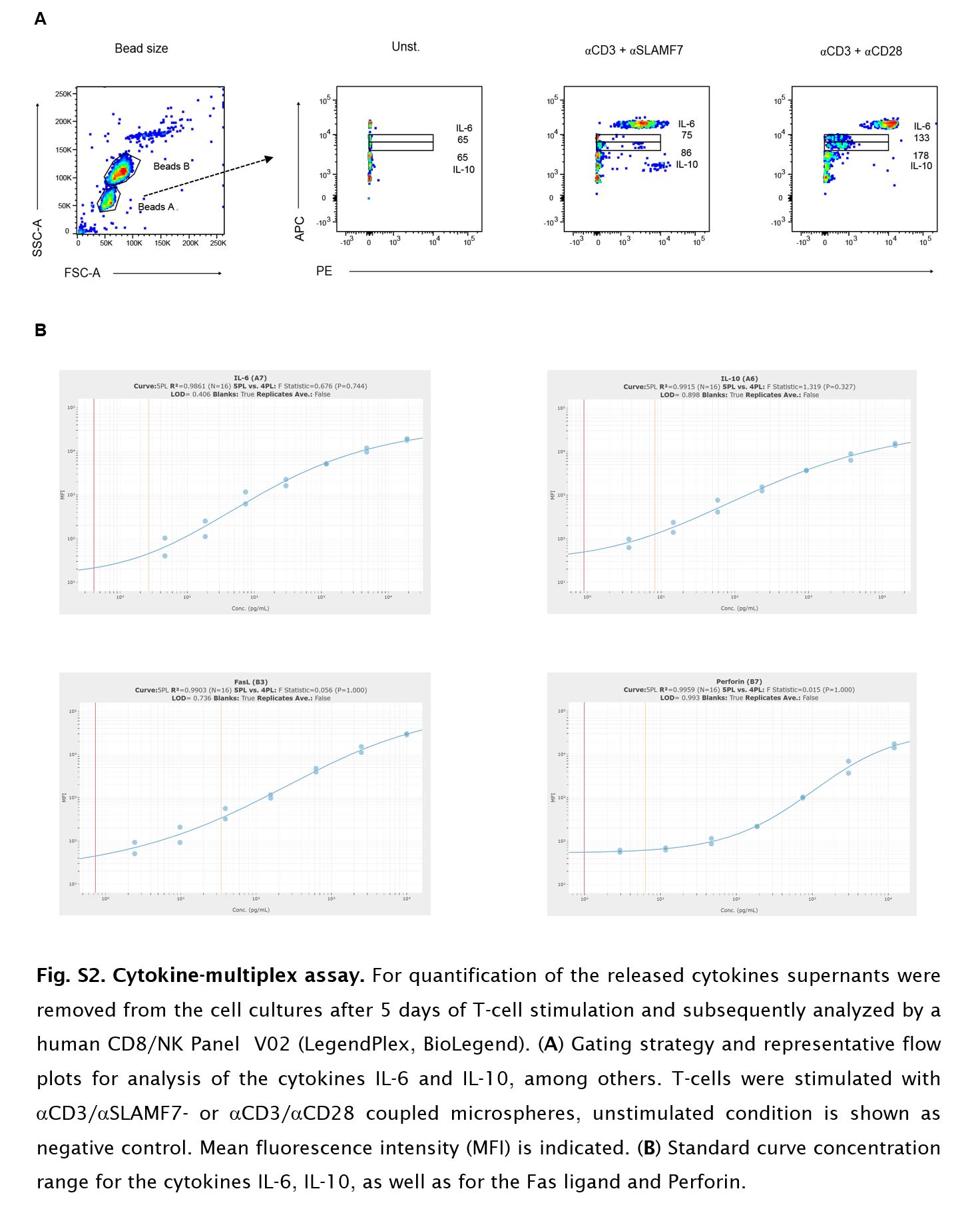

Supplement: Supplementary file 2 [file Image2.jpeg]

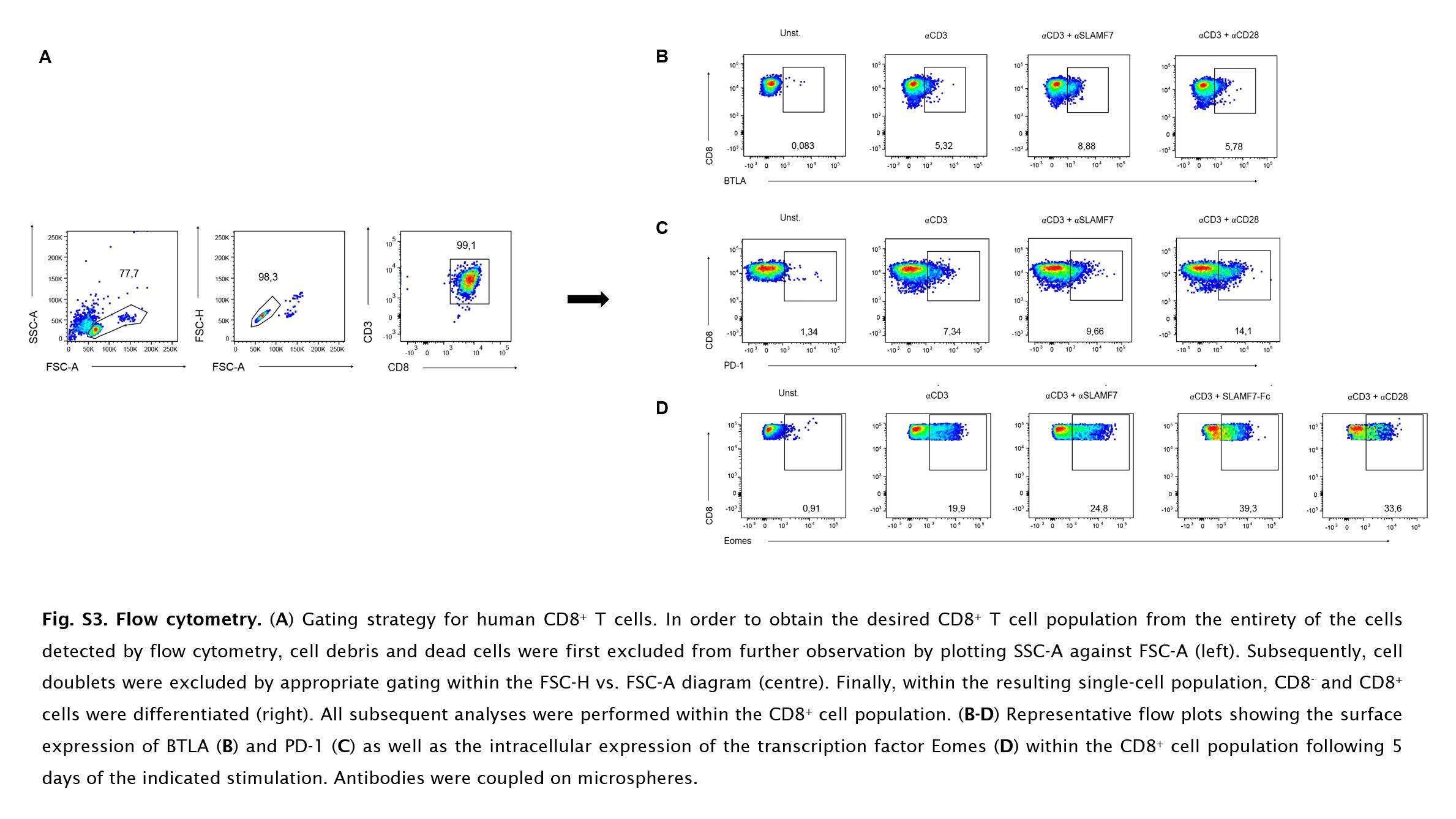

Supplement: Supplementary file 3 [file Image3.jpeg]

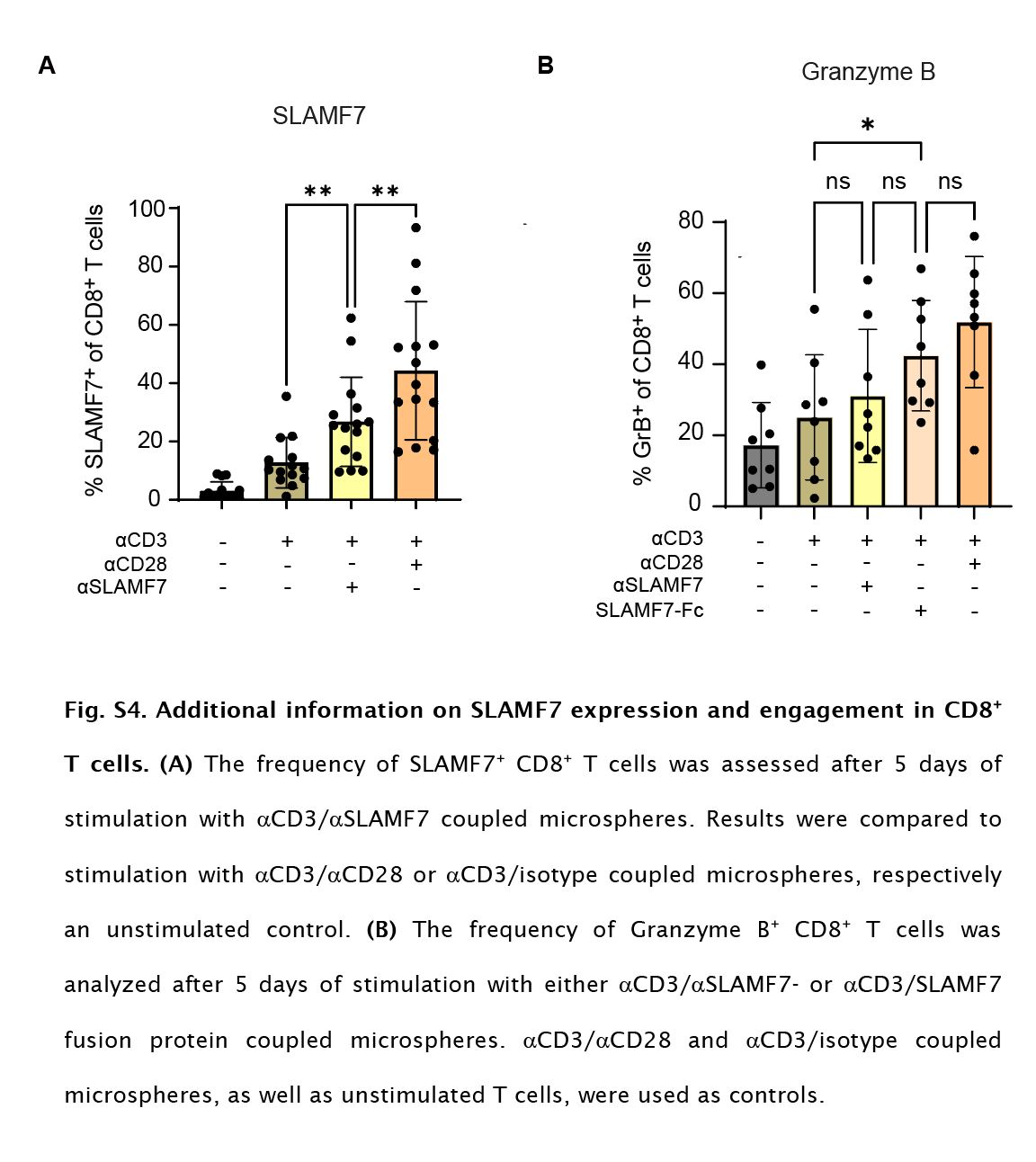

Supplement: Supplementary file 4 [file Image4.jpeg]

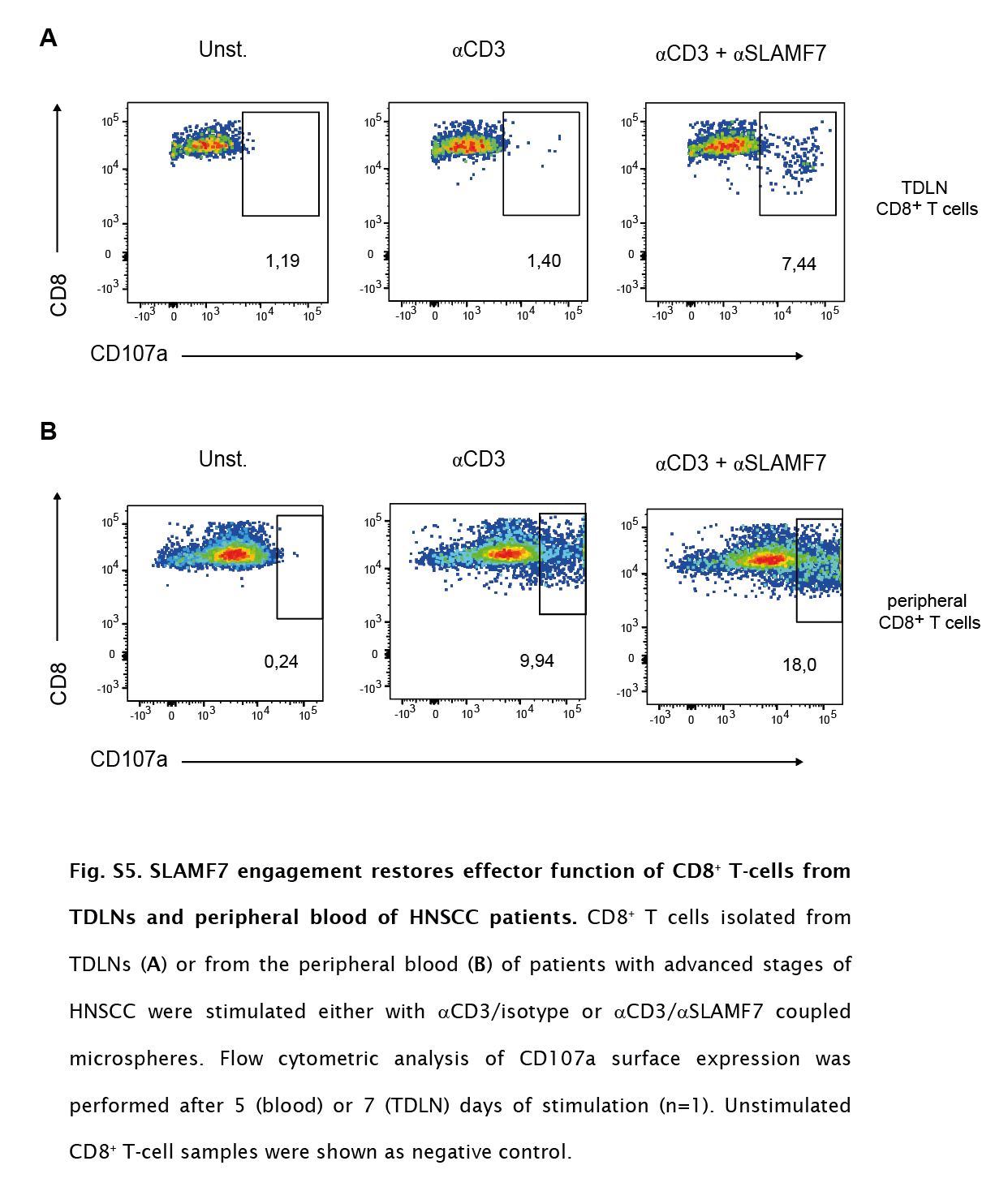

Supplement: Supplementary file 5 [file Image5.jpeg]
