# Supplementary material for: SLAMF7 (CD319) enhances cytotoxic T-cell differentiation and sensitizes CD8+ T cells to immune checkpoint blockade
Source: Front Immunol. 2025 Aug 20;16:1654374. doi: 10.3389/fimmu.2025.1654374 (PMC12405405; doi:10.3389/fimmu.2025.1654374)
Supplement: Supplementary file 6 [file Table1.docx]

**Supplementary Information**

**Supplementary Table 1A. Functional antibodies**

| Antibody | Clone | Species/Isotype | Company |
| --- | --- | --- | --- |
| αCD3 | HIT3α (Ultra-LEAF purified) | Mouse IgG2a, κ | BioLegend, San Diego, USA |
| αCD28 | CD28.2 | Mouse IgG1, κ | BioLegend, San Diego, USA |
| αSLAMF7 | 162.1 | Mouse IgG2, κ | BioLegend, San Diego, USA |
| SLAMF7-Fc | recombinant human | HEK293 - derived | R&D Systems, Minneapolis, USA |
| Isotype control (SLAMF7) | MPC-11 | Mouse IgG2b, κ | BioLegend, San Diego, USA |
| DimerX I human (HLA-A2:Ig fusion protein) | recombinant soluble | Mouse IgG1, λ | BD Biosciences, Franklin Lakes, USA |
| αPD-1 | EH12.1 | Mouse IgG1, κ | BD Biosciences, Franklin Lakes, USA |
| αPD-L1 | MIH1 | Mouse IgG1, κ | BD Biosciences, Franklin Lakes, USA |
| Isotype control (ICB) | 107.3 | Mouse IgG1, κ | BD Biosciences, Franklin Lakes, USA |

**Supplementary Table 1B. Fluorescence-labelled antibodies**

| Antibody | Clone | Laser | Species/Isotype | Company |
| --- | --- | --- | --- | --- |
| αBTLA | MIH26 | PE | Mouse IgG2a, κ | BioLegend, San Diego, USA |
| αCD14 | M5E2 | PE-Cy7 | Mouse IgG2a, κ | BD Biosciences, Franklin Lakes, USA |
| αCD25 | 2A3 | BV711 | Mouse BALB /c IgG1, κ | BD Biosciences, Franklin Lakes, USA |
| αCD69 | FN50 | PE-Cy7 | Mouse IgG1, κ | BioLegend, San Diego, USA |
| αCD137 | REA765 | FITC | Recombinant human IgG1 | Miltenyi Biotec, Bergisch Gladbach, Germany |
| αPD-1 | 29F.1A12 | APC-Cy7 | Rat IgG2a, κ | BioLegend, San Diego, USA |
| αSLAMF7 | 235614 | Alexa Fluor 700 | Mouse IgG2a, κ | BD Biosciences, Franklin Lakes, USA |
| αCD8 | SK1 | BV510 | Mouse IgG1, κ | BioLegend, San Diego, USA |
| αHLA-A*02 | BB7.2 | APC | Mouse IgG2, κ | BioLegend, San Diego, USA |
| αCD3 | SK7 | APC-Cy7 | Mouse IgG1, κ | BioLegend, San Diego, USA |
| αEomes | X4-83 | FITC | Mouse IgG1, κ | BD Biosciences, Franklin Lakes, USA |
| αT-bet | O4-46 | BV786 | Mouse IgG1, κ | BD Biosciences, Franklin Lakes, USA |
| αCD107a | H4A3 | APC | Mouse IgG1, κ | BioLegend, San Diego, USA |
| αGranzyme B | GB12 | PE | Mouse IgG1 | ThermoFisher Scientific, Waltham, USA |
| FcR blocking Reagent |  |  | Anti-human | Miltenyi Biotec, Bergisch Gladbach, Germany |

**Supplementary Table 2. ELISpot stimulation conditions.** The tables show the respective stimulation conditions, as well as the approximate number of T cells per well for ELISpot plates A and B, which are shown in Supplementary Figure S1.

**Plate A**

| Well-no. | Condition (microspheres) | Cells per well (200 µl) |
| --- | --- | --- |
| A-1 | unstimulated | 5.000 |
| B-1 | αCD3/isotype | 5.000 |
| C-1 | αCD3/αSLAMF7 | 5.000 |
| A-2 | αCD3/SLAMF7-Fc | 5.000 |
| B-2 | αCD3/αCD28 | 5.000 |
| C-2 | unstimulated | 5.000 |
| A-3 | αCD3/isotype | 5.000 |
| B-3 | αCD3/αSLAMF7 | 5.000 |
| C-3 | αCD3/SLAMF7-Fc | 5.000 |
| A-4 | αCD3/αCD28 | 5.000 |
| B-4 | unstimulated | 2.000 |
| C-4 | αCD3/isotype | 2.000 |
| A-5 | αCD3/αSLAMF7 | 2.000 |
| B-5 | αCD3/SLAMF7-Fc | 2.000 |
| C-5 | αCD3/αCD28 | 2.000 |
| A-6 | unstimulated | 2.000 |
| B-6 | αCD3/isotype | 2.000 |
| C-6 | αCD3/αSLAMF7 | 2.000 |
| A-7 | αCD3/SLAMF7-Fc | 2.000 |
| B-7 | αCD3/αCD28 | 2.000 |
| C-7 | - | - |

**Plate B**

| Well-no. | Condition (microspheres) | Cells per well (200 µl) |
| --- | --- | --- |
| A-1 | CEFX HLA-A2:Ig/isotype | 50.000 |
| A-2 | CEFX HLA-A2:Ig/isotype | 25.000 |
| B-1 | CEFX HLA-A2:Ig/αSLAMF7 | 50.000 |
| B-2 | CEFX HLA-A2:Ig/αSLAMF7 | 25.000 |
| C-1 | CEFX HLA-A2:Ig/SLAMF-Fc | 50.000 |
| C-2 | CEFX HLA-A2:Ig/SLAMF-Fc | 25.000 |
| D-1 | CEFX HLA-A2:Ig/αCD28 | 50.000 |
| D-2 | CEFX HLA-A2:Ig/αCD28 | 25.000 |
| E-1 | NY-ESO-1 HLA-A2:Ig/isotype | 50.000 |
| E-2 | NY-ESO-1 HLA-A2:Ig/isotype | 25.000 |
| F-1 | NY-ESO-1 HLA-A2:Ig/αSLAMF7 | 50.000 |
| F-2 | NY-ESO-1 HLA-A2:Ig/αSLAMF7 | 25.000 |
| G-1 | NY-ESO-1 HLA-A2:Ig/SLAMF-Fc | 50.000 |
| G-2 | NY-ESO-1 HLA-A2:Ig/SLAMF-Fc | 25.000 |
| H-1 | NY-ESO-1 HLA-A2:Ig/αCD28 | 50.000 |
| H-2 | NY-ESO-1 HLA-A2:Ig/αCD28 | 25.000 |

**Supplementary Table 3. Corrected p-values for multiple comparisons using Bonferroni-Holm test.** The p-values for which the Bonferroni-Holm correction results in a changed significance level are written in **bold** letters.

| Figure | 1C | | 2 A | | | |
| --- | --- | --- | --- | --- | --- | --- |
| comparison | CD25  x SLAMF7 | SLAMF7 x CD25 | αCD3 vs. αCD3/αSLAMF7 | | αCD3/αCD28 vs. αCD3/αSLAMF7 | |
|  |  |  | CD69 | CD137 | CD69 | CD137 |
| p-value | <0,0001 | <0,0001 | 0,0004 | 0,0007 | 0,0272 | 0,0139 |
| corrected p-value | <0,0001 | **<0,0002** | 0,0008 | 0,0007 | 0,0272 | 0,0278 |

| Figure | 2D | | | 2F | | |
| --- | --- | --- | --- | --- | --- | --- |
| comparison | BTLA | IL-6 | IL-10 | GrB | Eomes | T-bet |
| p-value | 0,0029 | 0,002 | 0,002 | <0,0001 | <0,0001 | 0,004 |
| corrected p-value | 0,0087 | 0,006 | 0,004 | **<0,0002** | **<0,0003** | 0,004 |

| Figure | 2E | | | | | | | |
| --- | --- | --- | --- | --- | --- | --- | --- | --- |
| comparison | αCD3 vs. αCD3/αSLAMF7 | | αCD3 vs. αCD3/SLAMF7-Fc | | αCD3/αSLAMF7 vs. αCD3/SLAMF7-Fc | | αCD3/αCD28 vs. αCD3/SLAMF7-Fc | |
|  | Eomes | T-bet | Eomes | T-bet | Eomes | T-bet | Eomes | T-bet |
| p-value | 0,0123 | 0,0356 | 0,068 | 0,006 | 0,0375 | 0,3505 | 0,827 | 0,2994 |
| corrected p-value | 0,0246 | 0,0356 | 0,068 | **0,012** | 0,075 | 0,3505 | 0,827 | 0,5988 |

| Figure | 2I | | 2J | | 3A | |
| --- | --- | --- | --- | --- | --- | --- |
| comparison | IFNγ | GrB | Perforin | FasL | CEFX | NY-ESO-1 |
| p-value | 0,0156 | 0,00365 | 0,0415 | 0,0005 | 0,0312 | 0,0021 |
| corrected p-value | 0,0156 | 0,0073 | 0,0415 | **0,001** | 0,0312 | 0,0042 |

| Figure | 3B | | | |
| --- | --- | --- | --- | --- |
| comparison | CEFX | | NY-ESO-1 | |
|  | IFNγ | GrB | IFNγ | GrB |
| p-value | 0,025 | 0,0021 | 0,0625 | 0,31805 |
| corrected p-value | 0,025 | 0,0042 | **0,125** | 0,6361 |

| Figure | 3E | | | |
| --- | --- | --- | --- | --- |
| comparison | IFNγ | | GrB | |
|  | αCD3 | αCD3/αSLAMF7 | αCD3 | αCD3/αSLAMF7 |
| p-value | 0,0233 | 0,0312 | 0,0267 | 0,0152 |
| corrected p-value | 0,0466 | 0,0312 | 0,0267 | 0,0304 |
